# Framing Climate Change Impacts as Moral Violations: The Pathway of Perceived Message Credibility

**DOI:** 10.3390/ijerph19095210

**Published:** 2022-04-25

**Authors:** Jialing Huang, Janet Z. Yang, Haoran Chu

**Affiliations:** 1 School of Media and Communication, Shenzhen University, Shenzhen 518060, China; 2 Department of Communication, University at Buffalo, The State University of New York, 329 Baldy Hall, Buffalo, NY 14260, USA; zyang5@buffalo.edu; 3 College of Journalism and Communications, University of Florida, Gainesville, FL 32611, USA; chu.h@ufl.edu

**Keywords:** climate change, moral frame, credibility, behavior, moral foundations theory

## Abstract

Climate change has been increasingly discussed in moral terms in public discourse. Despite the growing body of research on the effectiveness of moral frames in bridging the ideological divide, few studies have examined the role that perceived credibility, an important element of any persuasive appeal, plays in facilitating the framing effect. With the objective of further understanding how moral frames may engage individuals with different ideologies in climate change and refining climate change messaging strategies, two experimental surveys were conducted to examine the effects of moral violation frames on climate engagement. Specifically, a moderated mediation model was tested. The model posits that message credibility mediates the relationship between moral frames and policy support, as well as the relationship between moral frames and behavior intention. Moreover, political ideology moderated the indirect effects of message credibility. Based on moral foundations theory, seven messages were designed to activate individualizing and binding moral foundations. The results indicated that credibility consistently mediated the effects of the moral violation frame on climate engagement and that liberal-leaning individuals were more likely to perceive an individualizing frame as more credible than a binding frame. However, this difference was smaller among conservative-leaning individuals, with evidence for this moderated mediation model found only for policy support among college students. This study suggests that credibility is key for effective moral violations arguments of climate change.

## 1. Introduction

Climate change has significant impacts on public health, the global economy, and national security, which means that it unavoidably poses many moral challenges. Thus, it is not surprising that Pope Francis called climate change a “human abuse of God’s creation” in his encyclical letter and “a sin against future generations” in a public speech [1]. In the film “An Inconvenient Truth”, Al Gore also stated the following: “I don’t consider this [climate change] a political issue, I consider it to be a moral issue” [2]. These public discourses reflect the increasingly popular perception of climate change as a moral violation, that is, an event that violates moral standards (e.g., “to be fair”, “do no harm” [3]).

Despite the overwhelming evidence concerning climate change and its severe impacts on natural and human systems, the partisan divide in public opinion continues to widen [4]. Republicans tend to be skeptical of anthropogenic climate change, while the majority of Democrats express deep concern regarding this problem. Several explanations for this discrepancy have been proposed, including solution aversion [5] and partisan media exposure [6]. Scholars have also argued that this divide may be attributed to the different moral standards upheld by Republicans and Democrats [7]: Conservative-leaning Republicans tend to favor order, tradition, and protection of their in-groups, whereas liberal-leaning Democrats endorse compassion, equal opportunities, and the pursuit of communal goals, which are associated with support for climate change mitigation [8].

Therefore, communication scholars have argued that moral discourses may serve the goal of bridging this divide in public opinion, and such claims have found strong empirical support [9,10,11,12]. However, the mechanism underlying moral framing effects has been less explored. Previous research has mainly pointed to value-message congruency as a key explanation [13,14], suggesting that a moral frame is appealing to audiences who hold similar values as those proclaimed in the message. But is it also perceived as the “right” message? In other words, do people agree on the credibility of different moral frames, and more importantly, is that perception relevant to the messages’ persuasiveness? Considering that credibility is an important component of persuasive appeals [15], it is surprising that not much research on moral frames has examined such a factor. It is thus the goal of the current research to find out the role that credibility plays in explicating the effects of moral arguments. Since climate change information involves a great deal of uncertainty [16], focusing on the credibility of moral messages is crucial. The present research also explores the potential influences of political ideology on perceived credibility due to the well-documented partisan divide in climate beliefs [17]. By examining these questions, the present research aims to uncover what is driving the effects of moral frames, which can ultimately further the understanding of the persuasiveness of the increasingly popular moral framing of climate change. It can also provide practical implications for communication practitioners in refining climate communication strategies. Below, we begin with a review of the moral frames used for climate change by first focusing on the underlying framework–moral foundations theory.

### 1.1. Moral Foundations Theory

Moral foundations theory [18] asserts that humans have five innate moral foundations that guide their moral judgments. These foundations are *care* (i.e., concern for the suffering of others), *fairness* (i.e., concern for injustice and inequality), *loyalty* (i.e., concern related to obligations to in-group members), *authority* (i.e., concern regarding the social order and deference to authority), and *purity* (i.e., concern related to physical and spiritual contagion) [18]. Because care and fairness foundations are associated with individuals’ rights and welfare, they are also called “individualizing foundations”. In contrast, loyalty, authority, and purity foundations are pertinent to preserving social norms among an in-group, so these dimensions are referred to as “binding foundations” [19].

Individuals may prioritize certain moral domains in their moral judgment, depending on their cultural background, social-economic status, gender, religion, and political ideology [20]. Research has shown that among the five foundations, liberals are more sensitive to individualizing foundations. Conservatives, in contrast, do not show much variance across all foundations [19]. When comparing liberals and conservatives, liberals are more concerned about individualizing foundations than conservatives, whereas conservatives are more likely to endorse binding foundations than liberals [19]. Notably, while these findings suggest that liberals and conservatives differ in their level of endorsement of the five foundations, researchers later discover that when making a moral judgment on influential people, they rely on the same three sets of moral foundations (e.g., care, fairness, and purity), suggesting that the ideological differences may have been exaggerated by previous research [21].

The differences in moral intuitions, albeit may be smaller than what previous researchers expected, between these two groups may explain the partisan divide concerning the value of the environment and support for environmental protection [22]. The goal of protecting nature and future generations overlaps more with Democrats’ beliefs in care and fairness, and the solution to environmental problems often requires increased government regulations, which is contradictory to Republicans’ fundamental support for free-market economics [4,5]. As a result, liberal Democrats in general are more concerned about the environment than are conservative Republicans [23,24]. Research has also shown that liberals tend to view environmental protection as a moral issue, while conservatives do not [25]. Due to these discrepancies, developing moral frames that resonate with liberals as well as those that resonate with conservatives is a promising strategy that helps us reach these two groups effectively. In fact, research is accumulating on the moral framing of climate change.

### 1.2. Moral Frames for Climate Change

Framing is defined as a process used “to select some aspects of a perceived reality and make them more salient in a communicating text, in such a way as to promote a particular problem definition, causal interpretation, moral evaluation, and/or treatment recommunication” [26] (p. 52). Frames, as products of framing strategies, are organized central ideas of an issue that affect how people make sense of that issue [27]. Framing simplifies a complex topic by putting greater weight on certain considerations than on others [28]. Furthermore, frames influence people’s attitudes and behaviors more effectively when they align with pre-existing meaning structures and schemas established by personal experience [27]. For example, a message frame focusing on the collective benefits of environmental protection may have a greater impact on people who value self-transcendence and care about the welfare of others than on those who do not [29].

Over the years, climate change communication research has examined the effects of various frames, including social progress, economic development, and scientific uncertainty [28]. Increasingly, climate change has been discussed as a matter of right and wrong. For example, philosophers have described climate change as a “perfect moral storm” [30]. The United Nations called the task of slowing global warming a “moral imperative” [31]. In addition, many young Americans agree that climate change represents an ethical issue [14].

Given the increasing attention to the moral challenges presented by climate change, using moral arguments to motivate climate actions may be desirable. On the one hand, moral values exist universally and are believed to be innate within individuals [32]. A moral message about climate change could resonate with a wide group of audiences. On the other hand, climate change impacts are often portrayed in an abstract and distant manner [33]. Connecting climate change to personal morality can make it more local and relevant [34]. Moreover, because activating moral intuitions is powerful in motivating prosocial actions [35], it could work effectively in increasing pro-environmental behaviors. Due to these advantages, research has examined the effectiveness of moral frames in climate communication. For example, applying moral foundations theory, Wolsko et al. [12] asked participants to read a pro-environmental statement with an individualizing frame or one with a binding frame. Right-leaning participants who read the binding message expressed more pro-environmental beliefs than their counterparts who read the individualizing message, while left-leaning individuals were equally responsive to both messages. These results suggest that morality-congruent frames are more effective in communicating climate change to conservatives, a finding that is largely consistent with those of other studies (e.g., [10,11,25]). To engage conservatives, certain studies have framed climate change using language that reflects conservative moral values and religious beliefs, such as “protection of God’s creation” or “stewardship over the earth” [36], or emphasized the impact of climate change on national security [37,38]. The results of these studies have provided mixed findings concerning the effects of these moral frames.

Prior research examining moral frames has mainly focused on calls for actions related to particular moral foundations. Examples include the following: “You can make a difference by recycling because you know it is the right thing to do. Your action can help care for others…” [13] (p. 365) and “Demonstrate your respect by following the examples of your religious and political leaders who defend America’s natural environment. SHOW YOUR PATRIOTISM!” [12] (p. 10). To date, few studies have systematically examined the framing of climate change impact as a moral violation, that is, portraying it as a challenge to what we value, such as individual rights and group norms. Research has found that climate change has increased economic inequality, resulting in a strong decline in the annual income growth of hotter, poorer countries and an increase in that of wealthier, cooler countries over recent decades [39], which could be particularly alarming to individuals who strongly value fairness. Moreover, climate change is also closely related to environmental pollution, such as air and water pollution, soil contamination, and noise pollution, which may harm the purity of the natural environment [40]. Climate change can also cause difficulties for national security. For example, countries may fight for resources, and the number of refugees may increase as a result of extreme weather, shortages of food and water, and land loss due to rising sea levels [41], which presents a threat to binding foundations that focus on group benefits. Emphasizing these moral consequences of climate change may be more effective than merely advocating for individuals to uphold these principles because individuals tend to react more strongly to negative stimuli than to positive stimuli [42]. As such, it is the goal of this study to determine the role moral violation frames play in climate communication. Specifically, based on the conceptualization of morality in moral foundations theory [18], we focused on frames pertaining to violations of individualizing (e.g., concerns about human equality and health) and binding moral principles (e.g., concerns about border security, stewardship, and natural cleanliness).

### 1.3. The Perceived Credibility of Moral Frames

Most previous studies compare individualizing and binding frames conditional on the audiences’ ideologies, assuming that their persuasiveness is specific to whom they are speaking (e.g., [10,12,13]). Rarely have researchers examined whether individualizing and binding frames differ in their effects on general audiences. We argue that individualizing frames can outperform binding frames in general, and we examine credibility as an underlying mechanism. After all, credibility is a key determinant of a persuasive appeal. A considerable amount of research has indicated that audiences are more likely to be persuaded by strong arguments than weak arguments [15]. For climate communication research, credibility is even more important because of the skepticism surrounding climate change and contradictory messages competing in the risk information landscape [43]. Even for a message centering on normative beliefs (e.g., beliefs concerning what one should do), which are subjective in nature [44], they still need to establish credibility to make an impact. Therefore, the present research aims to discover whether credibility differentiates the impact of individualizing frames and binding frames.

Message credibility is defined as “an individual’s judgment of the veracity of the content of communication” [45] (p. 63). Important formative indicators of message credibility include messages that are complete, objective, expert, professional, concise, and well-presented [45]. There are reasons to believe that individualizing frames are more credible. First, prevalent messages pertaining to individualizing frames emphasize scientific arguments such as the claim that climate change poses a threat to human wellbeing and equality. As a result, such frame likely appears to be more professional and exhibit greater scientific expertise than binding frames that focus on personal beliefs such as harm to God’s creation or our loyalty to our motherland. Second, individualizing frames are more common in the media than are binding frames. Communication concerning climate change often pertains to individualizing values. A content analysis of public service announcements and newspaper op-eds suggested that the media communicate climate change primarily in terms of care (e.g., protecting the earth and human health) and fairness (e.g., the “climate debt” that wealthy countries owe to poor countries) [9]. The greater frequency of individualizing frames could make them appear more credible than binding frames due to mere exposure effects [46]. Thus, we propose Hypothesis 1, which examines the mediating role of credibility on policy support and behavior intention, two well-researched variables representing attitudinal and behavioral engagement concerning climate change:

**Hypothesis** **1a** **(H1a).** *Credibility mediates the effect of the moral frame on policy support, such that individualizing frames have a stronger indirect effect on policy support via credibility than do binding frames.*

**Hypothesis** **1b** **(H1b).** *Credibility mediates the effect of the moral frame on behavior intention, such that individualizing frames have a stronger indirect effect on behavior intention via credibility than do binding frames.*

### 1.4. The Moderated Mediation Effect of Political Ideology

The mediating role of credibility is likely conditional on political ideology. Specifically, we argue that being politically liberal enlarges the gap between individualizing frames and binding frames in terms of their persuasion effects for a couple of reasons. First is the different thought styles employed by liberals and conservatives. There is strong evidence suggesting that liberals tend to think more analytically than conservatives. In contrast, conservatives are more intuitive thinkers [47]. Moreover, liberals have a higher need for cognition and favor more complex messages [48]. As a result, liberals tend to rate stronger arguments as more persuasive, but conservatives do not differentiate between the persuasiveness of strong and weak arguments [49]. The seemingly objective statements in individualizing messages can be more congruent with liberals’ analytic mindset and argument preferences than binding messages, which results in liberals valuing messages with individualizing frames more highly. Indeed, empirical evidence supports that when thinking more analytically and thoroughly, individuals give greater value to individualizing moral foundations [50].

**Figure 1:**
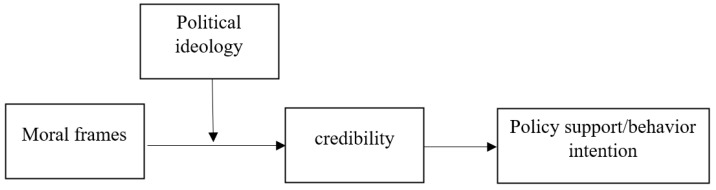
Hypothesized moderated mediation model.

At the same time, research suggests that individuals do not always aim for an accurate conclusion when processing information; they are also motivated by directional goals [51]. In the latter case, credibility assessment of new information can be a function of one’s information processing goals. For example, individuals tend to wish to maintain a connection to their in-group [52], which could bias their assessment of information based on their group values [53]. This directionally motivated reasoning related to climate change information is well documented (e.g., [51,54,55]). Because the individualizing frame is more congruent with liberals’ values [19], liberals are motivated to evaluate the message in a favorable way. In contrast, conservatives may be less motivated to do so. Indeed, research has shown that Republicans are less likely to be persuaded by science-based messages about climate change’s health impacts [55]. Moreover, liberals are more likely to express “feeling right” when encountering individualizing frames, whereas conservatives seem to have a penchant for binding frames [13]. This feeling could be misattributed to the quality of the message [56]. Taken together, we propose the following moderated mediation model (see Figure 1) and Hypothesis 2:

**Hypothesis** **2** **(H2).** *Political ideology moderates the strength of the mediated relationships between moral frames and policy support (H2a) and behavior intention (H2b) via perceived credibility, such that the mediated relationship is stronger in the context of a liberal political ideology than in the context of a conservative political ideology.*

## 2. Pilot Study

### 2.1. Method

#### 2.1.1. Sample and Procedure

To test the models, we conducted two survey experiments (i.e., a pilot and main study). The pilot study is a smaller-scale study that allows us to check on the procedures and provide initial evidence. For the pilot study, a total of 169 students from a large northwestern university in the United States were recruited and received course credit for their participation. An experimental survey where the experimental treatments were embedded in the survey was conducted, which allows us to examine the causal effects of moral violation frames. Participants were asked to view a randomly selected short message concerning the impact of climate change, pass an attention check, and then answer questions evaluating their opinions of the message and climate change. Participants were mostly female (97, 57.4%; *n*_male_ = 71, 42%) and white (95, 56.2%, *n*_African-American_ = 22, 13.0%, *n*_Asian_ = 34, 20.1%), with an average age of 20.7 (*SD* = 2.3). All procedures were approved by an Institutional Review Board.

#### 2.1.2. Stimuli

Due to the multi-dimensionality of moral foundation [18], to increase the content validity of our experimental stimuli, we manipulated moral violations of individualizing and binding foundations with seven messages concerning the impact of climate change. Each reflected one of the five moral foundations and presented common moral arguments in public discourse [57]. (1) Fairness: “global inequality” (*n* = 26) and “biodiversity loss” (*n* = 12); (2) Care: “human health” (*n* = 31) and “animal health” (*n* = 29); (3) Loyalty: “national security” (*n* = 22); (4) Authority: “God’s creation” (*n* = 32); (5) Purity: “water contamination” (*n* = 17). Because images can convey information quickly and effectively, to strengthen the manipulation and provide information complementary to the text, each message was paired with a relevant photograph. For example, a picture showing a flood destroying a crude house in Kiribati, an island country in which one in four people lives under the poverty line (Borgenproject.org, 2017), was displayed alongside the “global inequality” message (see Appendix A for all stimuli used in this research). We combined fairness and care messages into the “individualizing” condition (coded as 1, *n* =71), and we combined authority, loyalty, and purity into the “binding” condition (coded as 0, *n* = 98). As an introduction to the message, a brief definition of climate change was included.

#### 2.1.3. Measures

*Political ideology.* Eight items were adopted from Wolsko et al. [12] to measure political ideology. Participants indicated how much they were in favor of or against partisan policies such as more socialized health care and capital punishment (reverse coded) (*M* = 5.16, *SD* = 1.06, *α* = 0.82, ranging from 1 = *Strongly against* to 7 = *Strongly in favor*). A higher value indicated a more liberal ideology.

*Perceived credibility.* Participants rated the messages on three bipolar scales: 1 = inaccurate to 7 = accurate; 1 = inauthentic to 7 = authentic; and 1 = unbelievable to 7 = believable (*M* = 5.14, *SD* = 1.39, *α* = 0.91) [45].

*Policy support.* Six items were used to measure support for climate change mitigation policies [58] by inquiring into the extent to which participants supported or opposed mitigation policies. Sample items include the following: “Require automakers to increase the fuel efficiency of cars, trucks, and SUVs to 54.5 mpg” and “Simplify licensing procedures for companies in green technology businesses” (1 = *Strongly oppose* to 7 = *Strongly support*; *M* = 5.19, *SD* = 0.98, *α* = 0.80).

*Behavior intention.* Five items were adapted from [14] to measure mitigation behavior intention. Participants indicated the extent to which they agreed with statements such as “I intend to use only recyclable and reusable products from now on” (1 = *Strongly disagree* to 7 = *Strongly agree*). A composite was computed by averaging the five items (*M* = 4.67, *SD* = 1.28, *α* = 0.89).

### 2.2. Results

**Figure 2:**
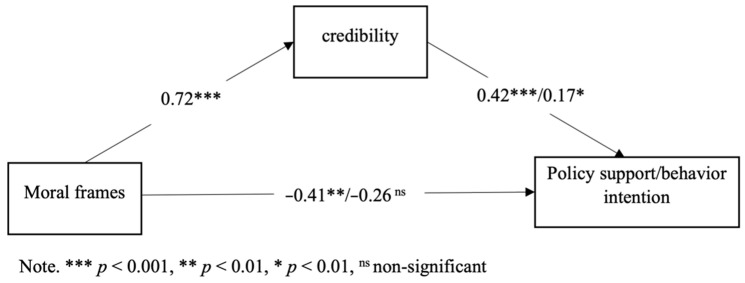
Mediation only model with standardized coefficients in the pilot study.

We used Hayes’s [59] PROCESS macro for SPSS to estimate the hypothesized model. Effects were estimated using 5000 bootstrap samples to create bias-corrected confidence intervals (C.I.s). To test H1, we ran PROCESS Model 4 (i.e., a mediation-only model). If zero is not within the 95% C.I. of the mediation index, then Hypotheses 1 is supported. Indeed, the results showed that the mediating effects of credibility were significant, as zero was not within the 95% C.I. The indirect effect of the moral frame on policy support was 0.30, bootstrap SE = 0.08, 95% C.I. = [.15, 0.47]. The indirect effect on behavior intention was 0.17, bootstrap SE = 0.08, 95% C.I. = [0.03, 0.35]. Individualizing frames were perceived to be more credible than binding frames, and credibility was significantly associated with policy support and behavior intention. See Figure 2. Therefore, H1 was supported.

To test Hypothesis 2, we used PROCESS Model 7 to see if the moderated mediation effects were significant. If zero is not within the 95% C.I. of the moderated mediation index, then Hypotheses 2 is supported. The results showed that it was significant for policy support: moderated mediation index = 0.11, 95% C.I. = [0.001, 218], as zero was not within the C.I. [59]. However, for behavior intention, the moderated mediation effect was not significant. The result suggested that political ideology moderated the mediating effects of credibility on policy support. In addition, the model explained 17% of the variance in credibility, *p* < 0.001, and 16% of the variance in policy support, *p* < 0.001 and 3% in behavior intention, *p* > 0.05.

In addition, political ideology was found to moderate the effect of the moral frame on credibility at a marginally significant level, B = 35, *p* = 0.05. Specifically, among more liberal individuals, the difference between the individualizing frame and binding frame in perceived credibility was larger. Moreover, higher credibility was associated with greater policy support and behavior intention, B = 0.30, *p* < 0.001, B = 0.17, *p* < 0.05.

**Figure 3:**
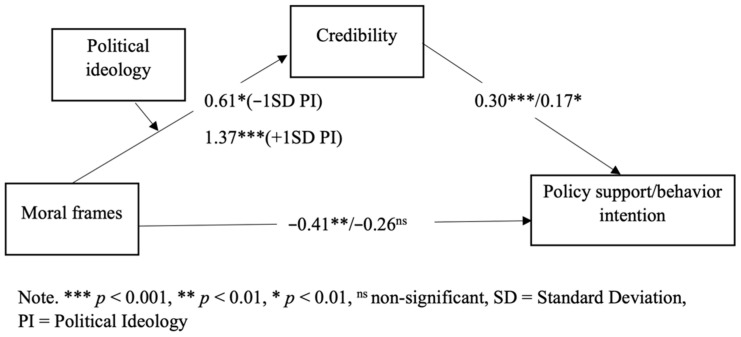
Mediated moderation model with unstandardized coefficients in the pilot study for the policy support variable.

Next, because the moderated mediation effect on political support was significant, we further examined the conditional indirect effect of the moral frame on policy support via political ideology at three values, including −1 SD below the mean, the mean, and 1 SD above the mean. The results showed that the conditional indirect effects of credibility were significant at all levels, and the effects were stronger for higher-level of political ideology. The results further confirmed the mediating role of credibility proposed in H1 and suggested that being more liberal enhanced the mediating effect of credibility in explaining the moral frame-policy support link. See Table 1 for the conditional indirect effects and Figure 3 for the model with coefficients. In summary, Hypothesis 2 was partially supported.

### 2.3. Discussion

The pilot study provides initial evidence supporting a moderated mediation model. We intend to validate the model with a larger and nonstudent sample recruited from Amazon Mechanical Turk (MTurk). Compared with American undergraduates, who are predominantly liberal, young, and highly educated, MTurk samples are more demographically diverse [60], approximating the US population more closely in terms of age, gender, race, and education [61]. Testing the model with a more diverse sample could provide additional evidence for the relationships identified in the pilot study. In the main study, to further explore the effect of moral violation frames on individual climate engagement, we also considered a new variable that is closely related to moral messaging–moral engagement, as described below.

#### Moral Engagement

While we have focused on one’s general attitudes toward policies and behaviors, another question that has yet to be answered pertains to whether moral frames evoke individuals’ feelings of moral obligation to act on climate change, a response that should be more closely induced by moral frames. Examining moral engagement is important because it is a key predictor of climate engagement [14] and it allows us to further validate the effect of moral violation frames. Moral engagement with climate change can be characterized by the extent to which one emphasizes the moral significance of actions, asserts the responsibility of various individuals or groups, highlights harmful effects, values victims, and encourages empathy [62]. One barrier to climate change communication is that, in reality, most people probably do not view themselves as morally responsible for climate change and instead blame others for climate change [63], which may be due to a sense of helplessness [64]. However, increasing moral engagement is important because this factor has been identified as an important antecedent of climate change mitigation intentions and an important mediator of climate change opinions and pro-environmental attitudes [14,65]. Framing climate change impacts as moral violations could be effective in increasing moral engagement. When people witness a moral transgression, they become more sensitive to their moral values and increase their involvement in actions that uphold those values [3]. Thus, in the main study, we examined the direct and indirect effects of moral violation frames on moral engagement by adding this factor as an outcome variable. Since right-wing individuals tend to be more morally disengaged from anthropogenic climate change than left-wing individuals [62], we continued to study the potential moderating role of political ideologies in these relationships.

## 3. Main Study

### 3.1. Method

#### 3.1.1. Sample

Two hundred and six participants (Mage = 37.56, *SD* = 10.82) were recruited from MTurk. Most participants were male (119, 57.8%; *n*_Female_ = 78, 37.9%), white (164, 79.6%; *n*_Asian_ = 15, 7.3%; *n*_Black_ = 11, 5.3%; other ethnicities constituted less than 5% of the sample), had a bachelor’s degree (114, 55.3%) and had an annual household income between $50,000 and $79,999 (79, 38.3%). Each participant was paid $1 for participating. Data were collected from 17 April 2021 to 24 April 2021.

#### 3.1.2. Stimuli and Measures

The same messages used in the pilot study were utilized, but the introduction was removed to emphasize the moral frames.

*Moral engagement.* Two measures were adapted from Leviston and Walker [65] to assess moral engagement. Participants were asked about the extent to which they agreed with the following two statements: “I feel a moral duty to do something about climate change” and “I feel it is my ethical responsibility to change my individual behavior to combat climate change” (1 = *Strongly disagree* to 7 = *Strongly agree*). These two items were averaged to create a composite.

Measures for all other variables were identical to those used in the pilot study. See Table 2 for correlation matrices among variables in both studies and the descriptive statistics for each variable in the main study.

### 3.2. Results

**Figure 4:**
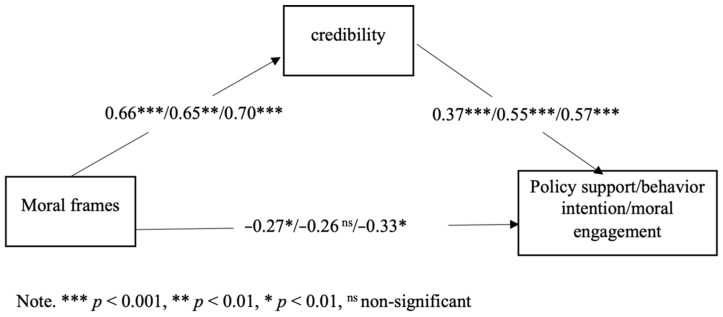
Mediation only model with standardized coefficients in the main study.

The same analytical procedure was employed. The results of the mediation-only model showed that the mediating effect of credibility was significant. The indirect effect of a moral frame on policy support was 0.25, bootstrapped SE = 0.09, 95% C.I. = [0.10, 0.43]. The indirect effect of moral frames on behavior intention was 0.36. bootstrapped SE = 0.11, 95% C.I. = [0.14, 0.59]. The indirect effect of a moral frame on behavior intention was 0.32, bootstrapped SE = 0.09, 95% C.I. = [0.15, 0.49], consistent with the pilot test; individualizing frames were perceived to be more credible than binding frames, and credibility significantly predicted policy support and behavior intention. Thus, Hypothesis 1 was again supported. See Figure 4 for the mediation-only model.

**Figure 5:**
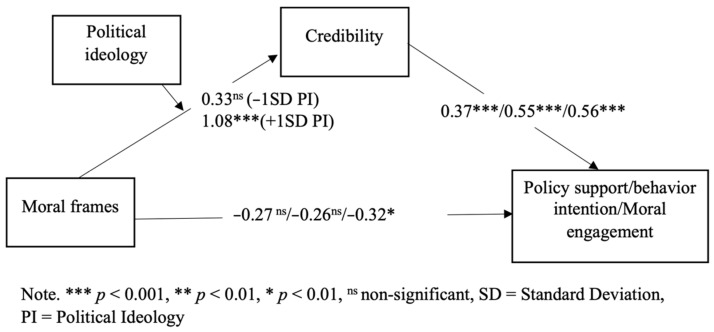
Moderated mediation model with unstandardized coefficients in the main study.

However, the results showed that the moderated mediation effects were not significant for all three outcome variables, as zero was within the 95% CI. Specifically, for policy support, the moderated mediation index = 0.11, 95% C.I. = [−0.035, 0.230]. For behavior intention, the moderated mediation index = 0.15, 95% C.I. = [−0.06, 0.36]. For moral engagement, the moderated mediation index = 0.17, 95% C.I. = [−0.05, 0.35]. The model explained 8% of the variance in credibility, *p* < 0.01, 23% of the variance in policy support, *p* < 0.001, and 30% in behavior, *p* < 0.001. See Figure 5.

For policy support and behavior intention, the interaction effect of political ideology and moral frames was significant only at the *p* < 0.10 level, B = 0.29, *p* = 0.06. In terms of moral engagement, the interaction effect was significant, B = 30, *p* < 0.05. Consistent with the pilot test, among individuals who were more liberal, the difference between the individualizing frame and binding frame in terms of perceived credibility was larger. Again, higher credibility was associated with greater policy support, behavior intention, and moral engagement. B = 0.37, *p* < 0.001. B = 0.55, *p* < 0.001, B = 0.57, *p* < 0.001.

## 4. General Discussion

This research set out to examine the persuasiveness of moral violation frames for climate change impacts among individuals with different political ideologies. It also examines the mediating role that perceived credibility plays with respect to the message effect on climate change engagement. The results consistently showed that moral frames affect climate engagement indirectly via credibility. Moreover, participants rated individualizing frames more credible than binding frames, especially among liberals. However, the moderated mediation effects on which the study focused were only observed among the student sample with respect to their policy support. Overall, although moral arguments are increasingly prevalent in climate change communication [34], our findings suggest that their potential effects may go beyond a mere value-message congruency effect.

### 4.1. Credibility Is an Important Component for Moral Frames

The significant mediation effect of credibility observed in the two studies suggests that across ideological groups, individualizing frames have an advantage over binding frames in increasing moral climate engagement because of the higher credibility. This relative advantage could be a result of both heuristic and deliberative information processing. For a heuristic judgment, the individualizing frames present a more accessible argument than binding ones. A content analysis of newspapers in Chile revealed that compared to conservative newspapers, liberal newspapers published twice as many articles regarding climate change and that their articles were twice as long, having four times as many illustrations [66]. Similarly, in the U.S., the climate change discourse is primarily carried out using liberal rhetoric [9]. The prevalence of individualizing language of climate change could make it more familiar for audiences. In addition, individualizing frames may be more salient than binding ones due to their relevance to the dominating moral intuitions. It is argued that care and fairness are the core moral values among all five foundations [67,68]. Indeed, empirical evidence suggests that liberals and conservatives both rely on care, fairness, and purity to judge the moral character of a person [21]. Thus, tagging on the core foundations, individualizing frames can be more salient (thus more accessible) than binding ones. For deliberative processing, individualizing frames can be functional because they are related to health impact and global equality, which seem more scientifically grounded topics than binding frames that often highlight personal beliefs. However, the claim needs further empirical validation. For example, it would be useful to conduct a content analysis between individualizing frames and binding frames to identify the number of scientific arguments in both types of messages.

Ultimately, the consistent significant mediation effects of credibility confirmed the importance of credibility for persuasive appeals [15]. In the context of the present research, credibility functions as a key mechanism in differentiating the effectiveness of individualizing and binding frames. Thus, while moral arguments are advocated by scholars mainly because they are relevant to different individuals’ moral intuitions [8,11,12], we cannot overlook the importance of “looking right” as a basic assessment for those arguments. To increase the credibility of moral frames, the literature has suggested that there are effective ways such as including valid sources [69] and increasing graphic interactivity [70].

### 4.2. The Role of Political Ideology in Credibility of Moral Frames and Climate Engagement

Despite that individualizing frames are overall perceived to be more credible, we acknowledge that the perception of a “right” message is conditioned on one’s political ideology. This is suggested by the marginally significant interaction effect between a moral frame and credibility link in both studies. This finding is consistent with previous research in that individuals with different political ideologies reacted differently to moral frames (e.g., [12,13]). The discrepancy may be explained by the different processing modes or distinct motives between liberals and conservatives. On the one hand, the presumably greater scientific evidence in individualizing frames can make them more appealing to liberals, who prefer analytic processing and strong arguments [47]. If so, it is the science in the messages, not the congruent value, that is driving the influence, and other factors such as the need for cognition, and religiosity likely covary with political ideology to influence the result. On the other hand, message evaluation can be motivated. The need to maintain consistent beliefs with their in-group may motivate conservatives to discredit the individualizing frames that do not reflect their group values and do not seem to come from an ingroup member (e.g., priest).

Did the importance of credibility in facilitating moral frames to influence climate engagement differ between individuals with different ideologies (i.e., moderated mediation effects)? In the present research, the answer is inconclusive. We only observed significant moderated mediation effects on policy support for the college student sample but not the MTurk one. The significant moderated mediation effect among students implies a boundary condition. Specifically, although both are liberal-leaning, MTurk samples tend to be more diverse than student samples [60]. Because college students are characterized as a “Weird” population- White, Educated, Industrialized, Rich, and Democratic [71], the results suggest that the moderated mediation effect is bounded by a homogenous sample. What’s more, the moderated effects have not been observed in behavior among the two samples, which we suspect that it is because motivating one to express support for mitigation policies at a collective scale is easier than getting them ready for actions at an individual level. However, these explanations need empirical verifications.

In sum, the findings suggest that although moral frames are a promising message strategy, it is vital to remain mindful of audiences’ preferences when using them. Specifically, we should emphasize the credibility of individualizing frames to liberal audiences, but on other merits of binding frames when communicating to conservative audiences. In this way, we can maximize the effects of moral frames and bridge the ideological divide in climate engagement.

### 4.3. Communicating Climate Change Impacts as Moral Violations

Most previous research on the use of moral frames for climate change has focused on the positive outcome of mitigation actions, with certain notable exceptions (e.g., [9]). Our study shows that framing the negative impacts of climate change in moral terms may also be effective by producing a sense of moral obligations. In this sense, our research resembles the loss-frame strategy used in health communication research [72,73]. In environmental communication research, loss frames have also been shown to be more effective in inducing positive reactions and generating stronger behavior intention than gain frames [74,75]. In this research, we only focused on moral violation frames, but it would be interesting to contrast gain vs. loss framing through a moral lens in future studies.

### 4.4. Limitations

This study faces certain limitations. First, the two samples were both liberal-leaning and are not representative of the population, which limits the external validity of our findings. As such, a more ideologically balanced and representative sample is needed for future research. In addition, the study was conducted in the United States because historically, it is the biggest carbon emitter in the world [76] and the political divide on climate engagement is substantial [17]. Thus, it is urgent to examine ways to engage its polarized citizens. However, future research should include non-US samples. Notably, our sample sizes in both studies were sufficient. Empirical estimates of the sample size needed to attain 0.80 power for a complex mediation model with a medium effect size using Monte Carlo simulations range from 110 to 220 [77]. The average effect size of all our endogenous variables (R^2^ = 0.16) is considered medium according to Cohen [78]. Second, our paper explored a limited set of moral frames. They are chosen because they represent prevalent moral arguments in public discourse [57]. Similar frames have been examined in previous research (e.g., [36,37,38,55]), which allows our findings to be connected to the larger body of literature. However, it is important to note that a diverse range of frames can be used to activate each moral intuition. There may be more effective messages to activate a specific foundation when communicating to specific groups. For example, while God could be considered an authoritative figure for religious individuals, others may see it as less relevant. Empirical evidence suggests that youth climate activists construct moral narratives in which younger generations, climate scientists, and the state are key agents of change [79], suggesting that other exemplars may resonate more with young people. Thus, future research should explore other potential candidates to represent individualizing and binding foundations. Third, the research did not feature a control group including participants who were not exposed to any moral frames. Future research should assess the persuasive effect of individualizing frames and binding frames against a message that does not particularly appeal to any of the moral foundations.

## 5. Conclusions

Climate change is the defining issue of our generation. To improve climate change communication, moral arguments have been increasingly popular. Against this backdrop, this study examined the persuasive effect of the moral violation frame in climate change messaging and investigated to what extent the message effects could be attributed to message credibility. We found that credibility contributed to the persuasiveness of moral violation arguments, especially for individualizing frames. Liberal participants perceived the individualizing frames to be more credible, while credibility mattered less for conservative participants.

These findings provide valuable theoretical and practical implications. In sum, the current research increases our understanding of the mechanism underlying the effects of moral frames by looking through the lens of credibility perception, a key concept in persuasion research and yet understudied in morality studies. Given that researchers have largely attributed the effects of moral frames to message-value congruency, our findings present a new direction for this line of research. Based on these findings, we also recommend that future research should continue to explore the messaging strategies based on moral angles that may be viewed as credible by liberals and reveal the mechanism that drives moral frames to work effectively among conservatives. With regards to practical implications, the current research provides valuable insights for communication researchers on creating credible moral messages for different audiences to maximize the persuasive effect.

## Figures and Tables

**Table 1:** Conditional Indirect Effects of Credibility on Outcome Variables.

	Level of Political Ideology	Index	BootStrap SE	BootStrap Lower Level CI	Bootstrap Upper Level CI
Policy support	Moderate (−1 SD)	0.180	0.089	0.026	0.374
	High (mean)	0.295	0.080	0.154	0.464
	Very High (+1 SD)	0.409	0.108	0.215	0.631

**Table 2:** Pearson Correlation Matrix.

**Pilot Study**								
	1	2	3	4	5			
1. Moral Frames	--							
2. Political Ideology	0.01	--						
3. Perceived Credibility	0.36 **	0.16 *	--					
4. Policy Support	−0.06	0.53 **	0.35 **	--				
5. Behavior Intention	−0.03	0.59 **	0.15	0.51 **	--			
**Main Study**								
	1	2	3	4	5	*M*	*SD*	*α*
1. Moral Frames	--							
2. Political Ideology	−0.05	--				4.38	1.24	0.80
3. Perceived Credibility	0.25 **	0.10 **	--			5.52	1.41	0.91
4. Policy Support	−0.01	0.37 **	0.47 **	--		5.45	1.10	0.83
5. Behavior Intention	0.04	0.09	0.54 **	0.70 **	--	4.94	1.38	0.90
6. Moral Engagement	0.03	0.29 **	0.61 **	0.78 **	0.77 **	4.43	1.53	0.82

Note. ** *p* < 0.01; * *p* < 0.05.

**Figure A1:**
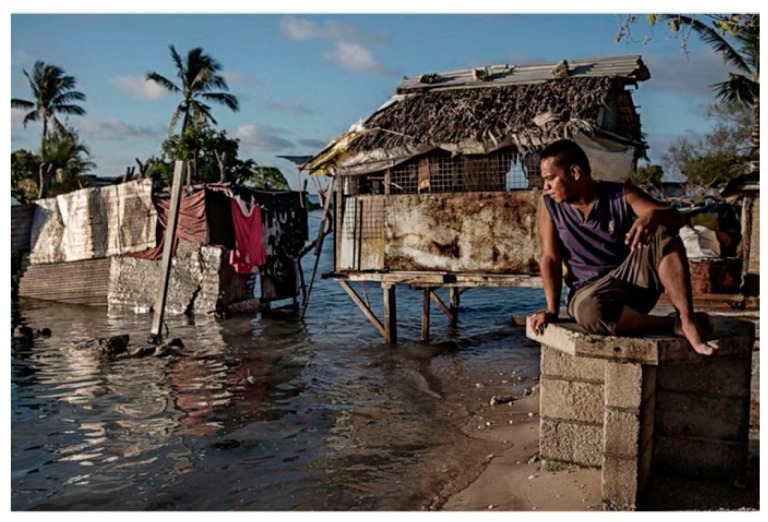
Stimulus Image for the Global Inequality Message.

**Figure A2:**
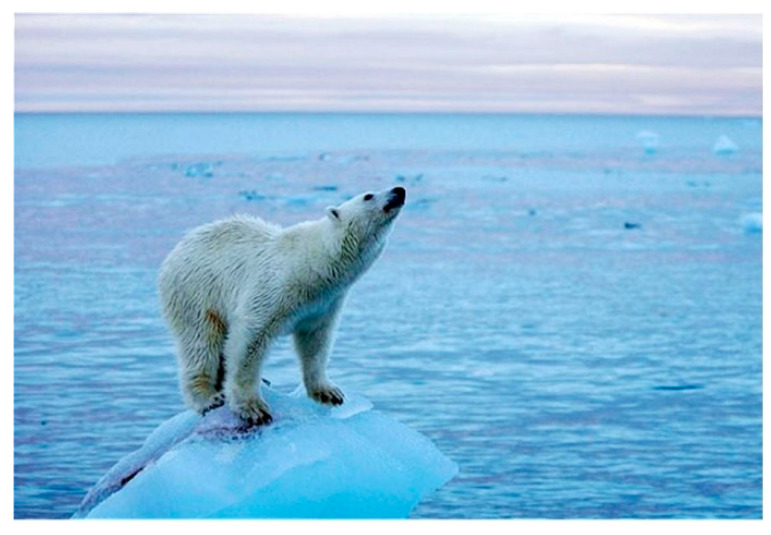
Stimulus Image for the Species Extinction Message.

**Figure A3:**
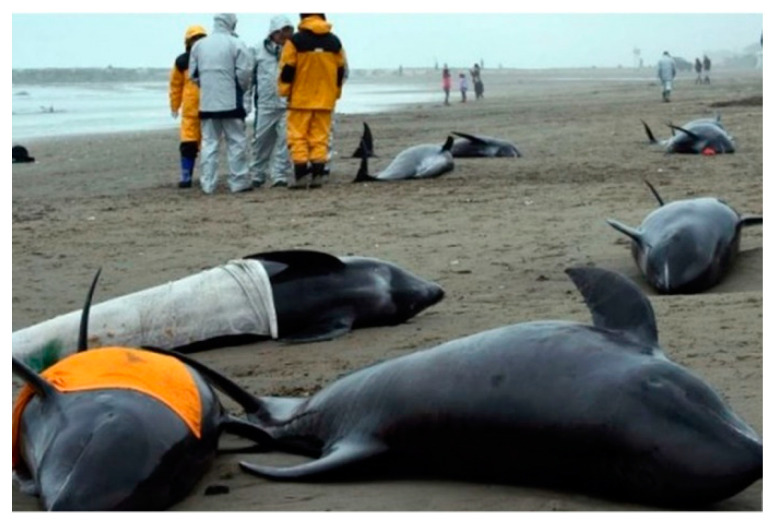
Stimulus Image for the Animal Health Message.

**Figure A4:**
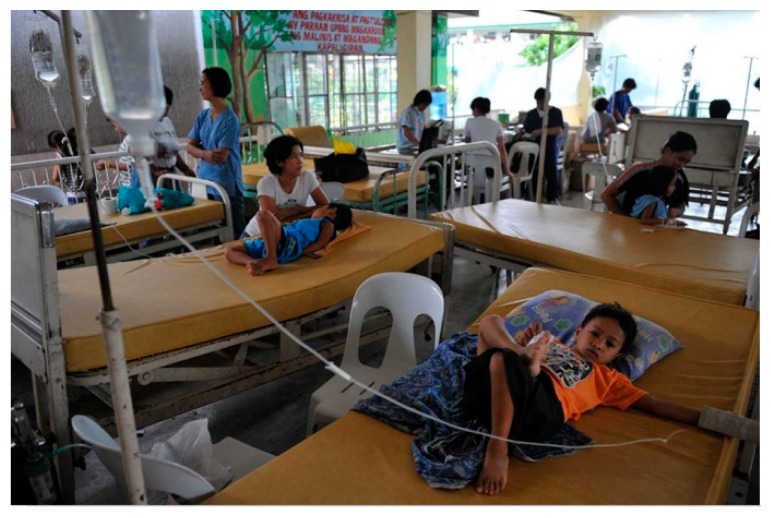
Stimulus Image for the Human Health Message.

**Figure A5:**
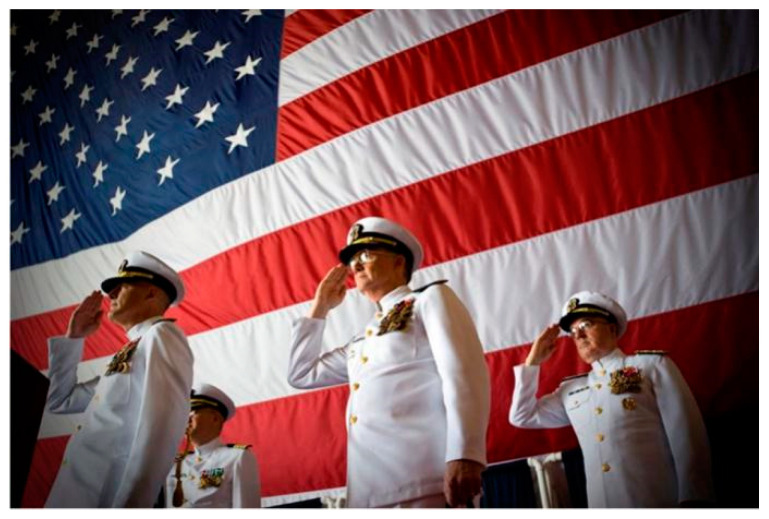
Stimulus Image for the National Security Message.

**Figure A6:**
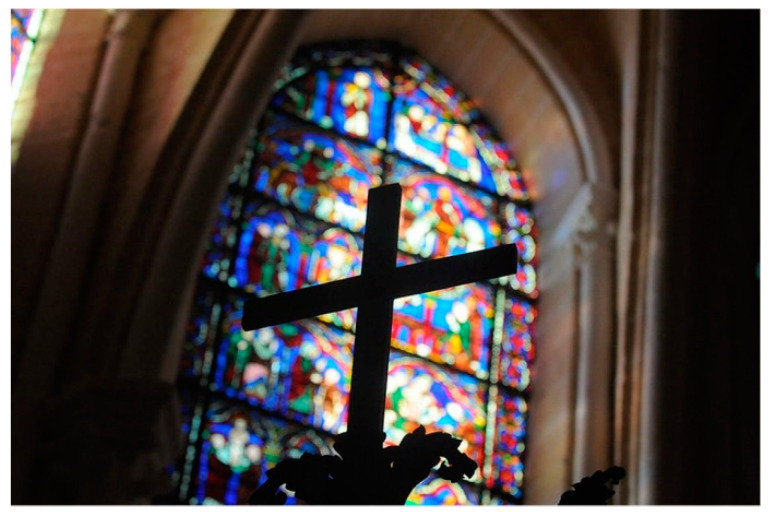
Stimulus Image for the God’s Creation Message.

**Figure A7:**
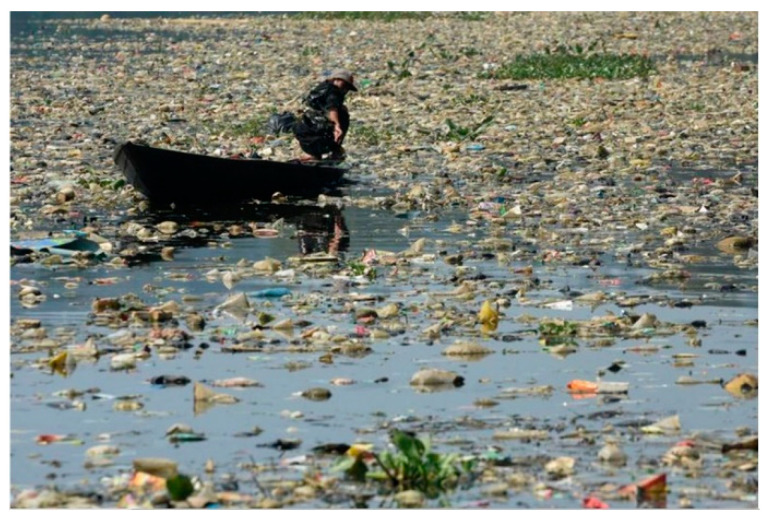
Stimulus Image for the Water Contamination Message.

## Data Availability

Publicly available datasets were analyzed in this study. This data can be found here: https://osf.io/jq7yx/?view_only=7df7772e716f4157aa56f14e72c77b9d (accessed on 2 February 2022).

## Appendix A. Stimulus Materials

### Appendix A.1. Global Inequality

Climate change worsens global inequality. The global wealth gap would be smaller today without climate change. Economic growth slowed in poor countries but accelerated in rich countries, because the richest countries are in cooler latitudes, while poor countries are disproportionately concentrated around the Equator, where even a slight increase in temperature can be devastating to their crop production and labor productivity.

### Appendix A.2. Species Extinction

For example, climate change accelerates species distinction. Climate change will cause the disappearance of nearly 8% of the current species. About one-third of corals, freshwater mollusks, sharks, and rays, one-fourth of all mammals, one-fifth of all reptiles, and one-sixth of all birds are heading towards extinction due to man-made climate change. As such, climate change poses a serious threat to world biodiversity.

### Appendix A.3. Animal Health

For example, climate change causes wildlife to suffer. It can alter disease states and animal health status and destroy their habitats. For example, increased temperature and frequency and intensity of heat waves may cause metabolic disruptions, oxidative stress, and immune suppression among livestock, leading to infections and death. Animals have to also change their breeding and feeding patterns in order to survive.

### Appendix A.4. Human Health

For example, climate change influences human health and disease in numerous ways. Some existing health threats will intensify and new health threats will emerge. The health effects include increased respiratory and cardiovascular disease, injuries and premature deaths related to extreme weather events, changes in the prevalence and geographical distribution of food- and water-borne illnesses and other infectious diseases, and threats to mental health.

### Appendix A.5. National Security

For example, climate change threatens national security. America’s domestic and international military bases are at risk as flooding and drought intensify. The impacts of climate change could result in armed conflicts over scarce food and water, putting U.S. troops in danger. The loss of Arctic sea ice may also lead to new conflicts with other Artic nations like Russia over national boundaries.

### Appendix A.6. God’s Creation

For example, climate change harms God’s creation. Nature is a magnificent book in which God speaks to us and grants us a glimpse of his infinite beauty and goodness. However, climate change gravely damages the environment of Sister earth. As Pope Francis notes in the encyclical letter, climate change is a crime human committed against the natural world and a sin against God.

### Appendix A.7. Water Contamination

For example, climate change contaminates our living environment. Climate change is making heavy intense downpours, droughts and rising water temperatures more common, which can alter the quality of our drinking and recreational water. More pollution is washed into our waterways: sediments, nitrogen from agriculture, disease pathogens, pesticides, and herbicides. It also increases the acidity of the ocean and allergens in the air.
